# The Protein Coded by a Short Open Reading Frame, Not by the Annotated Coding Sequence, Is the Main Gene Product of the Dual-Coding Gene MIEF1*[Fn FN2]

**DOI:** 10.1074/mcp.RA118.000593

**Published:** 2018-09-04

**Authors:** Vivian Delcourt, Mylène Brunelle, Annie V. Roy, Jean-François Jacques, Michel Salzet, Isabelle Fournier, Xavier Roucou

**Affiliations:** From the ‡Département de Biochimie, Université de Sherbrooke, Québec, Canada;; §Univ. Lille, INSERM U1192, Laboratoire Protéomique, Réponse Inflammatoire and Spectrométrie de Masse (PRISM) F-59000 Lille, France;; ¶PROTEO, Québec Network for Research on Protein Function, Structure, and Engineering, Québec, Canada

**Keywords:** Proteogenomics, Absolute quantification, Parallel reaction monitoring, Translation*, Gene Expression*, Knockouts*, Mass Spectrometry, Mitochondria function or biology, alternative translation, short ORF

## Abstract

Proteogenomics and ribosome profiling concurrently show that genes may code for both a large and one or more small proteins translated from annotated coding sequences (CDSs) and unannotated alternative open reading frames (named alternative ORFs or altORFs), respectively, but the stoichiometry between large and small proteins translated from a same gene is unknown. *MIEF1*, a gene recently identified as a dual-coding gene, harbors a CDS and a newly annotated and actively translated altORF located in the 5′UTR. Here, we use absolute quantification with stable isotope-labeled peptides and parallel reaction monitoring to determine levels of both proteins in two human cells lines and in human colon. We report that the main *MIEF1* translational product is not the canonical 463 amino acid MiD51 protein but the small 70 amino acid alternative MiD51 protein (altMiD51). These results demonstrate the inadequacy of the single CDS concept and provide a strong argument for incorporating altORFs and small proteins in functional annotations.

According to the traditional view of protein synthesis, each protein-coding gene harbors a single annotated open reading frames (ORF)^1^ or coding sequence (CDS) encoding a canonical protein. However, genes contain more than one ORF, and the longest ORF is generally designated as the canonical CDS in genome annotations (1). In eukaryotes, alternative splicing results in the production of several mRNAs and the translation of different isoforms, in addition to the canonical protein. Hence, the translational output of a protein-coding gene is currently concealed to a canonical protein and one or several isoforms.

This concept was recently disproved by two modern approaches to the accurate measurement of translation, ribosome profiling and proteogenomics. Ribosome profiling maps the regions of the transcriptome which are actively translated with nucleotide resolution (2). Proteogenomics approaches use customized protein databases and mass spectrometry (MS)-based proteomics to detect translated proteins (3–10). Both methods have revealed prevalent translation of ORFs outside of annotated CDSs and of out-of-frame ORFs (altORFs) (2, 11). These findings call into question the concept of the single CDS in eukaryotic mRNAs (12). In addition, they also highlight the need to redefine translated sequences (13), modernize functional genome annotations with shorter ORFs (14), and reassess the translation output of protein coding-genes by considering smaller proteins in addition to larger canonical proteins. The cellular stoichiometry of a canonical protein *versus* a small protein encoded in the same gene and their respective concentrations is unknown. Yet, proteins are the primary effectors of biological processes and deciphering the function of a gene in health and disease requires accurate characterization of its products.

The mitochondrial elongation factor 1 gene or *MIEF1* also termed *SMCR7L/MiD51*, localized at the Chr22q13.1 locus, codes for a mitochondrial receptor of Drp1, a GTPase which functions in mitochondrial fission (15–17).

Ribosome profiling and proteogeomics studies recently demonstrated the translation of a stable 70 amino acid protein product encoded in a altORF localized in the 5′UTR (Fig. 1*A*) (3, 18–24). Thus, *MIEF1* is a prototypical gene coding for both a large and a small protein. For simplicity, we termed this novel protein “alternative-MiD51” or altMiD51. Remarkably, both proteins are localized at the mitochondria. MiD51 is an outer mitochondrial membrane protein (17) whereas altMiD51 is located at the mitochondrial matrix (24) and both are involved in mitochondrial fission (17, 24). AltMiD51 has also been reported to be a new assembly factor of the mitochondrial ribosome and implicated in its biogenesis (25).

Here, we employ a targeted proteomics approach based on AQUA peptides to reliably quantify the absolute amount of MiD51 and altMiD51 in two human cell lines and one human tissue, and thus we establish an improved map of the translational output of *MIEF1/SMCR7L/MiD51* by directly measuring the final protein products.

## EXPERIMENTAL PROCEDURES

### 

#### 

##### Experimental Design and Statistical Rationale

In this study, our aim is to determine the stoichiometry of two distinct proteins encoded within the gene *MIEF1*, the canonical protein MiD51 and altMiD51. AltMiD51 is a 70 amino acids protein coded by a short ORF in the 5′UTR region. We expect to unveil the stoichiometry of the two proteins using targeted proteomics and stable isotope labeled synthetic peptides. To determine proteotypic peptides, we first performed AP-MS DDA experiments on both proteins. Peptides were then validated in overexpressed and endogenous conditions using targeted proteomics. Selected peptides were tested for coefficient of variation (CV) using technical replicates and were validated if their CV fell below 20%. One stable isotope peptide for each protein was purchased and tested for linearity in a peptide mixture using two technical replicates at various concentrations. Both peptides showed a linearity range between 40 amol and 250 fmol. Absolute quantification experiments were then conducted in two cell lines (three biological replicates each) and colon tissue (three technical replicates), in MiD51 knockout HeLa cells and altMiD51 knockout HeLa cells (three biological replicates each). Protein amounts were compared using Welch's two samples t-tests.

##### Tissue Collection and Ethics

Normal human colon tissue sample was obtained from the Biobanque des maladies digestives du CRCHUS. Patient gave informed consent for the banking and use of tissue sample. The ethic review board at the Centre intégré universitaire de santé et de services sociaux de l'Estrie - Centre hospitalier universitaire de Sherbrooke (CIUSSS de l'Estrie - CHUS) approved the use of this sample for this study. Briefly, following resection for colorectal cancer, the colon was washed thoroughly and normal tissue sampling was performed at more than 10 cm of the tumor, within a region confirmed by a pathologist to be uninvolved by tumor cells (H&E staining). Fresh sample was flash frozen in liquid nitrogen within 30 min of surgical resection.

##### Cell Culture

Cells were grown in Dulbecco's Modified Eagle Medium (DMEM, Wisent, St-Bruno, Quebec, Canada) supplemented with 10% fetal bovine serum (FBS, Wisent) and antibiotic-antimycotic mixture (Wisent). Cells were mycoplasma free (routinely tested). For transfections, cells were grown in 100 mm Petri dishes until 80% confluent and were transfected by adding 10 μg of plasmidic DNA in 2 ml of FBS/antibiotics-free DMEM and 10 μl of GeneCellIn (Eurobio, Les Ulis, France) and let to grow for 24 h before cell lysis. For parallel reaction monitoring (PRM) experiments, cells were grown in 60 mm Petri dishes until about 80% confluent.

##### DNA Constructs

DNA constructs were generated by Gibson assembly (26) of synthetic DNA (Gblocks, IDT, Skokie, Illinois) using the NEBuilder HiFi DNA Assembly Cloning Kit (New England BioLabs, Ipswich, Massachusetts) according to manufacturer's recommendation. DNA blocks of C-terminally LAP-tagged (27) MiD51 and GFP-tagged altMiD51 were inserted separately into pcDNA 3.1(-) expression vector (Thermo Fisher Scientific, Waltham, Massachusetts). The context construct was built on the assembly of the full 5′ region containing the altMiD51 coding sequence with a C-terminal 2 FLAG tag and the canonical MiD51 coding sequence with a C-terminal HA tag (transcript NM_019008.4) into pcDNA 3.1(-) expression vector. DNA sequences were controlled by sequencing.

##### Mitochondrial Extracts

For Western blot analysis of HeLa and CRISPR-Cas9 HeLa clones as well as PRM optimizations, mitochondrial extracts were performed according to (28) with minor modifications. Cells were grown into three 100 mm dishes until 80% confluent, rinsed twice with PBS 1× and collected using a cell scrapper. Cells were pelleted by centrifugation at 500 × *g* for 10 min at 4 °C. Supernatant was discarded and cells were suspended in mitochondrial buffer (mito-buffer : 210 mm mannitol, 70 mm sucrose, 1 mm EDTA, 10 mm HEPES-NaOH, pH 7.5, 2 mg/ml Bovine Serum Albumin (BSA), 0.5 mm PMSF and EDTA-free protease inhibitor (Thermo Fisher Scientific)) and disrupted by passage through a 25G1 0.5 × 25 needle syringe 15 consecutive times on ice followed by a 3 min centrifugation at 2000 × *g* at 4 °C. Supernatant was collected and the pellet was resuspended in mitochondrial buffer. The breakage procedure was repeated four times. All four supernatants containing mitochondria were again passed through syringe needle in mito-buffer and cleared by centrifugation for 3 min at 2000 × *g* at 4 °C. Supernatants were collected and centrifuged for 10 min at 13,000 × *g* at 4 °C to pellet mitochondria. Pellets were washed twice with BSA-free mitochondrial buffer and pooled. Final mitochondrial pellet was lysed in SDS buffer (4% SDS, Tris-HCl 100 mm pH 7.6). After sonication, protein content was assessed using BCA assay (Thermo Fisher Scientific).

### Mass Spectrometry Sample Preparation

#### 

##### Preliminary Affinity-purification (AP)

Cells were rinsed twice with cold PBS 1X and lysed with 1 ml of AP-buffer (NP-40 0.5%, Tris-HCl 50 mm pH 7.5, NaCl 150 mm, EDTA-free protease inhibitor 1×). Lysate was cleared by centrifugation (2000 × *g*, 5 min) and supernatant was collected. GFP-Trap agarose beads (ChromoTek, Planegg-Martinsried) were conditioned with three consecutive PBS 1× washes followed by three AP-buffer washes. Lysate supernatant was mixed with beads and incubated at 4 °C for 18 h on a rotating device. Beads were then washed 3 times with AP-buffer and 5 times with 50 mm NH_4_HCO_3_ (ABC). Digestion was performed on beads by adding 1 μg of trypsin (Promega, Madison, Wiscosin) in 100 μl ABC at 37 °C overnight. Digestion was quenched with formic acid to a final concentration of 1% and supernatant, containing peptides, was collected. Beads were then washed once with acetonitrile/water/formic acid (1/1/0.01 v/v) and pooled with supernatant. Peptides were dried using a speedvac, desalted using a C18 Zip-Tip (Millipore Sigma, Etobicoke, Ontario, Canada) and resuspended into 25 μl of 1% formic acid in water prior to MS analysis.

##### PRM Experiments

For mitochondrial extracts, mitochondrial pellet was lysed using SDS buffer as described above. For whole cell lysates, cells were rinsed twice with cold PBS 1× and lysed using SDS buffer. Tissue sample were homogenized using a TissueRuptor (Qiagen, Toronto, Ontario, Canada) in SDS buffer. Lysates were sonicated to reduce viscosity followed by a 5 min centrifugation at 14,000 × *g* to discard debris and insoluble parts. Protein content was assessed using BCA protein assay (Thermo Fisher Scientific). A total of 100 μg of protein and 1 μg of recombinant Glutathione S-transferase (GST, *Schistosoma japonicum*) were reduced by adding dithiothreitol to a final concentration of 50 mm and incubated 15 min at 55 °C. Lysates were prepared according to the filter aided sample preparation protocol (FASP) with minor modifications (29). Lysates were diluted with 500 μl of 8 m urea solution and transferred into a 3 kDa centrifugation device (Amicon Ultra, Millipore Sigma) and centrifuged for 30 min at 14,000 × *g*. After one 8 m urea wash and centrifugation, samples were diluted with 200 μl of 50 mm iodoacetamide in 8 m urea and left at room temperature in the dark for 30 min. Samples were centrifuged and washed 3 times with 8 m urea. Buffer was then exchanged for 50 mm ABC with three consecutive 200 μl washes. The final retentate was digested overnight at 37 °C with 1 μg of trypsin (Gold, Promega) in 40 μl ABC and AQUA (30) peptides (pepoTec Ultimate, Thermo Fisher Scientific). Tryptic peptides were collected by filter centrifugation followed by three ABC washes and centrifugation. Peptide-containing filtrate was concentrated using a speedvac and then acidified by formic acid to a final concentration of 1%. Peptides were desalted using a C18 Zip-Tip and dried using a speedvac.

##### Calf Intestinal Phosphatase Treatment

Peptides were dephosphorylated using calf intestinal phosphatase (CIP) according to (31). Briefly, 5 μg of desalted peptides were solubilized with 10 units of CIP (New England Biolabs) in 50 μl of CIP buffer (100 mm NaCl, 50 mm Tris-HCl, 10 mm MgCl_2_ and 1 mm DTT; pH 7.9) and incubated at 37 °C for 2 h. The mixture was acidified by adding trifluoroacetic acid (TFA) to a final concentration of 0.5%. Peptides were desalted using a C18 Zip-Tip (Millipore Sigma), dried and solubilized with 25 μl of 1% formic acid in water.

### nanoLC-MS/MS Analysis

#### 

##### Instrument Setup

A total of 12 μl of peptide mixture was loaded onto a trap column (Acclaim PepMap100 C18 column, 0.3 mm id × 50 mm, Thermo Fisher Scientific) at a constant flow rate of 4 μl/min. Peptides were separated in a PepMap C18 nano column (75 μm × 50 cm, Thermo Fisher Scientific) using a 0–35% gradient (0–215 min) of 90% acetonitrile, 0.1% formic acid at a flow rate of 200 nL/min followed by acetonitrile wash and column re-equilibration for a total gradient duration of 4 h with a RSLC Ultimate 3000 (Thermo Fisher Scientific, Dionex). Peptides were sprayed using an EASYSpray source (Thermo Fisher Scientific) at 2 kV coupled to a quadrupole-Orbitrap (QExactive, Thermo Fisher Scientific) mass spectrometer.

##### Affinity Purification and MS Analysis (AP-MS)

For preliminary AP-MS of GFP-tagged constructs, the mass spectrometer was used in data dependent acquisition mode (DDA). Full-MS spectra within a *m*/*z* 350–1600 mass range at 70,000 resolution were acquired with an automatic gain control (AGC) target of 1e6 and a maximum accumulation time (maximum IT) of 20 ms. Fragmentation (MS/MS) of the top ten ions detected in the Full-MS scan at 17,500 resolution, AGC target of 5e5, a maximum IT of 60 ms with a fixed first mass of 50 within a 3 *m*/*z* isolation window at a normalized collision energy (NCE) of 25. Dynamic exclusion was set to 40 s.

##### Data-dependent Protein Identification of AP-MS Samples

Mass spectrometry RAW files were searched with Andromeda (32), search engine implemented in MaxQuant 1.5.5.1 (33). Trypsin/P was set as digestion mode with a maximum of two missed cleavages per peptides. Oxidation of methionine and acetylation of N-terminal were set as variable modifications. Carbamidomethylation of cysteine was set as fixed modification. Precursor and fragment tolerances were set at 4.5 and 20 ppm respectively (defaults settings). Files were searched using a target-decoy approach (34) against Uniprot-Human 03/2017 release (35) and GST (92,949 entries) at a 1% false discovery rate at peptide-spectrum-match, peptide and protein levels. Peptides sequences were recovered from MaxQuant output files.

##### PRM Method Refinement

Developed and applied PRM analyses were Tier 2 assays. A first PRM method was determined with a large number of peptides in order to discriminate peptides that were detectable in overexpression as well as endogenous conditions in mitochondrial extracts. Peptide unicity was first checked using neXtProt peptide uniqueness checker (36). MS/MS spectra were then manually inspected and peptides with highest MS intensities, absence of miscleavage and high identification scores were selected for preliminary PRM peptide evaluation. The peptide list consisted in 30 mass over charges corresponding to unique peptides of MiD51 (11 peptides), altMiD51 (4 peptides), HSP60 (4 peptides) and GST (5 peptides) at various charge states. Method consisted in a Full-MS spectra acquisition with an AGC target of 3e6, maximum IT of 70 ms and a resolution of 70,000 followed by an unscheduled targeted-MS2 method with an AGC target of 5e5 ions, maximum IT of 130 ms, resolution of 17,500 with a 2 *m*/*z* isolation window and a NCE of 27.

A second method was used to evaluate the signal recovered after CIP treatment on endogenous mitochondrial extracts. The peptide list consisted in 11 mass over charges based on previous PRM experiments corresponding to peptides of MiD51 (3 peptides), altMiD51 (2 peptides), HSP60 (3 peptides) and GST (3 peptides). Method consisted in a Full-MS spectra acquisition with an AGC target of 3e6, maximum IT of 70 ms and a resolution of 70,000 followed by an unscheduled targeted-MS2 method with an AGC target of 5e5 ions, maximum IT of 150 ms, resolution of 17,500 with a 2 m/z isolation window and a NCE of 27. All method optimization files were processed using Skyline (37).

##### High Sensitivity PRM

For endogenous CV analysis in whole cell extracts and mitochondrial extracts as well as absolute quantification experiments, mass spectrometer was set for highest sensitivity according to (38). Method consisted into a Full-MS spectra acquisition with an AGC target of 3e6, maximum IT of 70 ms and a resolution of 70,000 followed by an unscheduled targeted MS2 method with an AGC target of 1e6 ions, maximum IT of 250 ms resolution of 70,000 and a NCE of 27. Isolation list contained one peptide from altMiD51, one for MiD51 and their AQUA standards, one peptide from GST spike-in as well as one peptide from HSP60 which were used as sample processing controls.

##### High Sensitivity PRM Sample Analysis

Mass-spectrometry RAW files were analyzed using Xcalibur 2.2 (Thermo Fisher Scientific) by measuring area of each peptide monoisotopic transitions within a 3 ppm mass precision window. For AQUA peptide calibration curves, internal standards were spiked into a HeLa digest and analyzed with high sensitivity PRM in conditions described above. For each peptide, five precursor-to-fragment transitions starting from N terminus within a mass deviation of 3 ppm were assessed for linearity and CV analysis, considering that a transition with a CV below 20% at a given concentration is quantifiable. For endogenous CV analysis, most quantifiable precursor to fragment transitions were measured for each peptide within a 3-ppm precision window, and two replicates were compared. For absolute quantification experiments, protein concentration was determined by comparing the ratio of the endogenous peptide to spiked-in AQUA standard and its concentration with the same precursor to fragment transitions within a 3-ppm mass precision window. Peptide ratios were kept below 25. Spectral similarity was controlled by importing RAW files into Skyline and peptides were validated if their spectral contrast angles (39) or ratio dot products were close to 1 as well as their retention times matching AQUA standards.

The mass spectrometry proteomics data have been deposited to the ProteomeXchange Consortium via the PRIDE (40) partner repository with the data set identifier PXD008147.

### CRISPR-Cas9-mediated MiD51 and altMiD51 Knockout (KO)

#### 

##### Knockouts Clonal Cell Generation

CRISPR-Cas9-mediated MiD51 and altMiD51 KO HeLa cells were generated according to (41) with minor modifications. Briefly, sgRNAs were designed using the Broad Institute sgRNA Designer (CRISPRko) tool (http://portals.broadinstitute.org/gpp/public/analysis-tools/sgrna-design, (42)) and confirmed with the CCTOP tool (http://crispr.cos.uni-heidelberg.de/, (43)). CRISPR-Cas9 related oligonucleotides are described in Table I. The sgRNA inserts, containing an extra G in 5′ required for the U6 RNA polymerase III promoter, were prepared by annealing the top and bottom oligos (Table I) and cloned into the pSpCas9(BB)-2A-GFP plasmid (Addgene #48138, Cambridge, Massachusetts, (41)). The resulting plasmids were verified by sequencing. Enrichment for Cas9–2A-GFP expressing cells and isolation of clonal cell populations were performed 24 h after transfection by single-cell FACS sorting. The initial validation of genome editing was done by Mismatch-cleavage assay using T7 Endonuclease I (NEB), GenElute Mammalian Genomic DNA Miniprep Kit (Millipore Sigma) with missmatch assays primers (Table I). Cells edition was confirmed by Western blotting (Fig. 3) and by sequencing PCR amplicons derived from the target sites.

**Table I:** Oligonucleotide sequences used for CRISPR-Cas9 genome editing experiments

	Oligonucleotide sequences for altMiD51 knock out	Oligonucleotide sequences for MiD51 knock out
Genomic target site	5′-TGGAGCCGAGAGGCGGTGCT-3′	5′-CGCTGGCAGTTAAGCGGGTA-3′
Top oligonucleotide	5′-CACCGAGCACCGCCTCTCGGCTCCA-3′	5′-CACCGTACCCGCTTAACTGCCAGCG-3′
Bottom oligonucleotide	5′-AAACTGGAGCCGAGAGGCGGTGCTC-3′	5′-AAACCGCTGGCAGTTAAGCGGGTAC-3′
T7 endonuclease 1 mismatch assays primers	5′-GGGGTCTCTGGAACTTGGAT-3′ 5′-TCCTTTTCTCGGTCCCTTGC-3′	5′-GGTCCCAGTACTTATGGCCG-3′ 5′-CCACGCAGAAAATCTCAGGG-3′

##### Characterization of Heterozygote MiD51 KOs

Genomic DNA was amplified using MiD51 mismatch assays primers with primer extension allowing its insertion into linearized (EcoRI, BamHI, New England Biolabs) pcDNA 3.1(-) expression vector *via* Gibson assembly as mentioned above. Plasmids were purified and sequenced.

##### Western Blotting

For each sample, 50 μg of mitochondrial protein extract was mixed 1/1 (v/v) with Laemmli buffer (4% SDS w/v, 20% glycerol v/v, Tris-HCl 100 mm pH 6.8, 5% β-mercapto ethanol v/v) and heated at 95 °C for 5 min. For altMiD51, proteins were separated in a 4% stacking/15% acrylamide-bisacrylamide (29/1 w/w) resolving SDS-PAGE for one hour at 200 V constant voltage using a glycine-buffer. For MiD51, proteins were separated in a 4% stacking/10% acrylamide-bisacrylamide (49.5% T, 3% C) resolving tricine SDS-PAGE (15, 44) gel (16 × 18 cm) for 18 h using 0.2 m Tris-HCl pH 8.9 as anode buffer and 0.1 m Tris-HCl, 0.1 m tricine, 0.1% SDS pH 8.25 as cathode buffer (25 mA constant current). Proteins were transferred onto polyvinyldiene difluoride membranes. The membranes were blocked with 5% milk supplemented Tris-buffered saline 0.2% Tween-20 (TBST). Membranes were probed with a custom anti-altMiD51 rabbit antibody (Proteintech, Rosemont, Illinois, see below), a polyclonal anti MiD51 rabbit antibody (Proteintech 20164–1-AP) and a mouse monoclonal anti-mitochondrial HSP 70 antibody (MA3028, Thermo Fisher Scientific) at 4 °C overnight. The membrane was then washed three times with TBST and probed with goat anti-mouse (sc-2005, Santa-Cruz Biotechnology, Mississauga, Ontario, Canada) or goat anti-rabbit-conjugated horseradish peroxidase antibodies (7074S, Cell Signaling Technology, Danvers, Massachusetts).

##### AltMiD51 Antibodies

Rabbit polyclonal anti-altMiD51 antibodies were raised against the full-length 70 amino acids recombinant altMiD51 protein and affinity purified (Proteintech).

##### Statistics

All graphics and statistics were made using R (45) 3.3.2 and ggplot2 (46) 2.2.1 or higher.

##### Cross Validation with Elongating Ribosomes

Ribo-Seq coverage of both ORFs were extracted from GWIPS (47) for HEK and HeLa cells. All nucleotides of both ORFs were considered as mappable using Umap track of UCSC Genome Browser (48) and ribosome densities were compared between altMiD51 and MiD51 ORFs.

## RESULTS

### 

#### 

##### Determination of MiD51 and altMiD51 Proteotypic Peptides

**Fig. 1.**
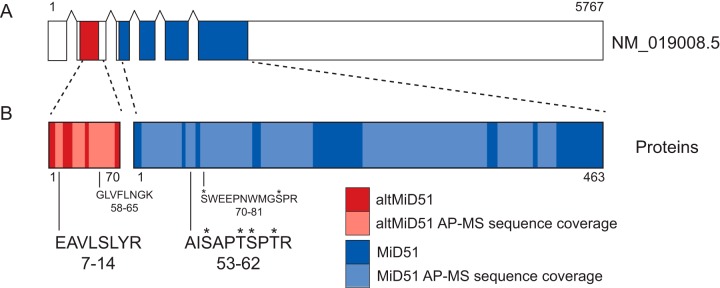
**Schematic representation of human *MIEF1* RefSeq variant 1 mRNA and altMiD51 and MiD51 proteins.** *A*, Human *MIEF1* includes 11 exons (RefSeq, GRCh38.p7). The mRNA variant 1 (NM_019008.5) shown here contains 6 exons. The CDS (blue) is shared between exons 3 to 6. AltMiD51 ORF is localized within exon 2, annotated as a non-coding exon. *B*, Sequence coverage in AP-MS experiments is represented in light colors. Proteotypic peptides sequence and positions (a.a.) are shown. EAVLSLYR and AISAPTSPTR peptides were selected for absolute quantification. *, known phosphorylated residue (phosphosite.org, (63)).

In addition to the canonical CDS (Consensus CDS CCDS13995; RefSeq NM_019008.5; Ensembl ENST00000325301) and associated protein (RefSeq NP_061881, UniProt Q9NQG6; Ensembl ENSP00000327124), human *MIEF1* contains a functional and recently annotated altORF (GenBank HF548110) coding for a small protein (Uniprot L0R8F8; GenBank CCO13821.1; Ensembl ENSP00000490747) (Fig. 1*A* and 1*B*). Thus, *MIEF1* is clearly a prototypical dual-coding gene for which the absolute quantification of the large and small protein products is unknown. We evaluated the ability of MiD51 and altMiD51 to generate proteotypic peptides after trypsin digestion. Proteotypic peptides are specific for each protein and they must be consistently detected with excellent quality precursor and fragment mass transitions (49–52). To facilitate the detection of specific tryptic peptides for both proteins, we used affinity purification coupled with mass spectrometry. MiD51^GFP^ and altMiD51^GFP^ were independently overexpressed in HeLa cells. Both proteins were affinity purified and analyzed via data-dependant (DDA) nano capillary liquid chromatography mass spectrometry (nanoLC-MS/MS). Several proteotypic peptides were detected for a total sequence coverage of 68.6% and 71.9% for altMiD51 and MiD51, respectively (Fig. 1*B* and supplemental Data S1). After manual evaluation (see method section), best quality proteotypic peptides were selected for parallel reaction monitoring (PRM) optimization (38, 53).

##### PRM Optimization for MiD51 and altMiD51 Proteotypic Peptides

Selected proteotypic peptides were then validated in low-sensitivity PRM experiments in 3 kDa FASP processed samples (29). Indeed, altMiD51 is a small protein of 70 amino acids and a low M.W. cut-off is necessary to ensure protein retention during sample preparation. Because both MiD51 and altMiD51 are mitochondrial proteins (24), mitochondria were isolated from mock-transfected cells and from cells transfected with a cDNA containing both the CDS coding for MiD51 and the native 5′UTR containing the altORF coding altMiD51 (RefSeq transcript NM_019008.4). Two proteotypic peptides for each protein were detected in these mitochondrial extracts (supplemental Fig. S1). Signal intensity for endogenous mitochondrial HSP60 peptide shows that the protein concentration of mock and transfected mitochondrial extracts were similar, and that the intensity difference for altMiD51 and MiD51 peptides between mock-transfected and altMiD51/MiD51-transfected samples did not result from differences in mitochondria preparation.

As MiD51's most intensely detected peptide (AISAPTSPTR) bore known phosphosites (Fig. 1*B*), a second PRM method including a dephosphorylation step using calf intestinal phosphatase (CIP) was implemented (31). A fraction of MiD51 was phosphorylated because CIP treatment resulted in a 20% increase in intensity for AISAPTSPTR (supplemental Fig. S2). The efficiency of CIP treatment was validated with two known HSP60 tryptic phosphorylated peptides, VGGTSDVEVNEK and VTDALNATR (phosphosite.org) with an increase in intensity of 103 and 133%, respectively. The intensity of a non-phosphorylated HSP60 peptide did not change significantly. AltMiD51 peptides are clearly nonphosphorylated because CIP treatment did not change significantly their intensity (supplemental Fig. S2). Based on these results, we selected peptides EAVLSLYR and AISAPTSPTR for absolute quantitation of altMiD51 and MiD51, respectively.

Finally, the precision of the most sensitive PRM method across different samples was estimated with the measure of the coefficient of variation (CV) on mitochondrial and whole cell extracts. Indeed, a CV below 20% is required for absolute quantification (54). The CVs were systematically below 20%, indicating that both mitochondrial and whole cell extracts were suitable for quantification (supplemental Fig. S3). Even though peptide intensities are higher in mitochondrial extracts, we decided to use whole cell lysates for absolute quantification of altMiD51 and MiD51 as their preparation does not involve cell fractionation, with the risk of variable mitochondrial recovery.

##### AltMiD51 and MiD51 Protein Abundances

Two synthetic stable isotope-labeled peptides for absolute quantification (AQUA) (30), EAVLSLYR and AISAPTSPTR, were spiked into the protein sample after trypsin digestion from HeLa cells and analyzed *via* PRM (Fig. 1*B*). A total of 5 y ion transitions starting from the most N-terminal amino acid were measured and both peptides displayed at least one quantifiable transition within a range of 40 amol - 250 fmol and a CV < 20% (supplemental Fig. S4).

**Fig. 2.**
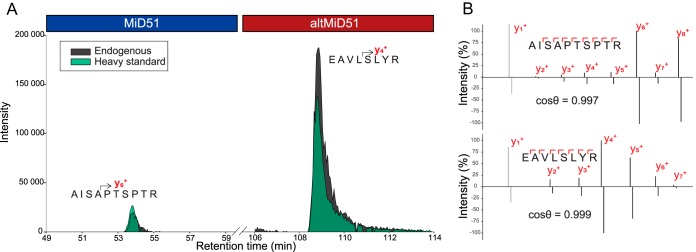
**Identification and quantification of altMiD51 and MiD51.** *A*, Extracted fragment-ion transition chromatograms of MiD51 ([AISAPTSPTR+2H]^2+^ → y_6_^+^) and altMiD51 ([EAVLSLYR+2H]^2+^ → y_4_^+^) peptides in HeLa cells. *B*, Spectral contrast angle analysis of endogenous peptides (top) and stable isotope labeled synthetic peptides (bottom) extracted from supplemental Fig. S5.

Absolute quantification PRM experiments were performed by spiking AQUA peptides with trypsin into the digestion mixture as described by (30). After desalting and dephosphorylation with CIP treatment, the resulting peptides were processed using a high sensitivity PRM method (38). For each peptide, retention times for the corresponding native and AQUA species as well as spectral contrast angles or ratio dot product (39) were controlled to ensure correct identification (Fig. 2*A*, 2*B* and supplemental Fig. S6–S12). The absolute amount of native peptides were thus determined (supplemental Data S2).

##### CRISPR-Cas9-mediated Independent Inactivation of altMiD51 or MiD51

**Fig. 3.**
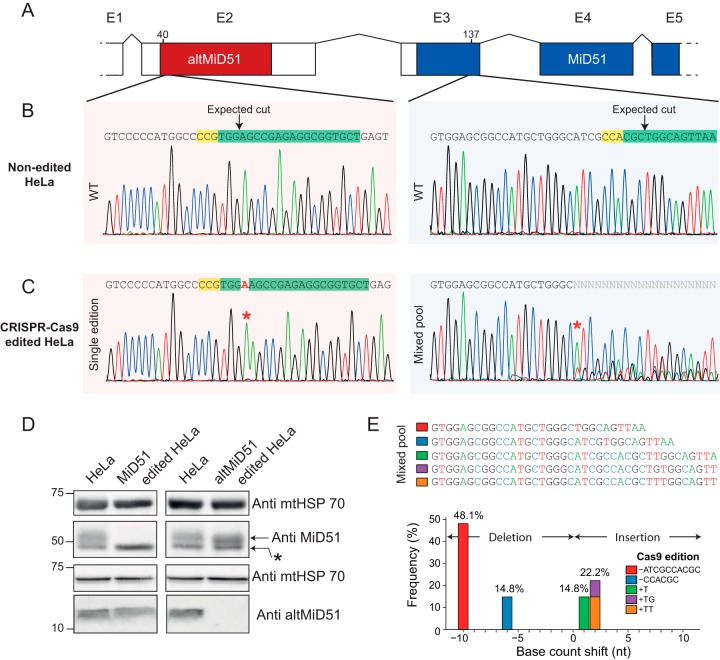
**CRISPR-Cas9 editing of genomic altMiD51 and MiD51.** *A*, Schematic representation of CRISPR-Cas9 experiments strategy. For clarity, only 5 exons are shown. Alt-MiD51 within exon 2 is shown in red. MiD51 coding sequence (blue), overlaps exons 3, 4 and part of exon 5. *B*, Genomic sequences around the programmed cut sites in non-edited HeLa cells and corresponding sequences. PAM sites are highlighted in yellow, and the genomic sequences targeted by the guide RNAs are highlighted in green. The programmed cut sites are also shown, at nucleotide 40 in exon 2 and nucleotide 137 in exon 3. *C*, Genomic sequence around the programmed cut sites in CRISPR-Cas9-edited HeLa cells. In the altMiD51-edited clone, a 1 bp A/T insertion (labeled in red, and red star above the electropherogram) occurred at the cut site. In the MiD51edited clone, a mixture of different sequences are detected 10 nucleotides upstream the programmed cut site (red star), indicating the presence of different alleles. *D*, Mitochondrial extracts from non-edited, MiD51-edited and altMiD51-edited HeLa cells were lysed and analyzed by Western blotting with antibodies against mtHSP70, MiD51 and custom altMiD51 antibodies, as indicated. *E*, CRISPR-Cas9 MiD51 knock-out sequence analysis. Sequences are aligned with electropherogram of panel c. * refers to a nonspecific target of MiD51 antibodies (17).

As this is the first absolute quantification of a large and small protein encoded by two independent ORFs in the same gene, it is important to show that absolute amounts of MiD51 and altMiD51 are partially or completely obliterated by inactivating their respective coding sequences. Experimental modulation of altMiD51 expression independently of MiD51 expression using a RNAi-based knockdown approach is impossible because both proteins are coded by the same gene, and both coding sequences are present in the same transcripts. This is a general challenge for the study of small and large proteins coded in the same gene (14). Thus, we implemented a CRISPR-Cas9 approach to independently prevent the expression of either altMiD51 or MiD51 (Fig. 3*A* and 3*B*) (41, 55).

Genome-edited clonal cell lines were validated by sequencing the targeted genomic region. The sequence of the PCR-amplified altMiD51 genomic region confirmed the homozygous 1 bp insertion of a A/T at position 40 of exon 2, at the Cas9 cleavage site (Fig. 3*C*). For MiD51, the sequence electropherogram of the PCR-amplified genomic region showed overlapping peaks (Fig. 3*C*), indicating the presence of heterozygous mutations in the different alleles.

**Fig. 4.**
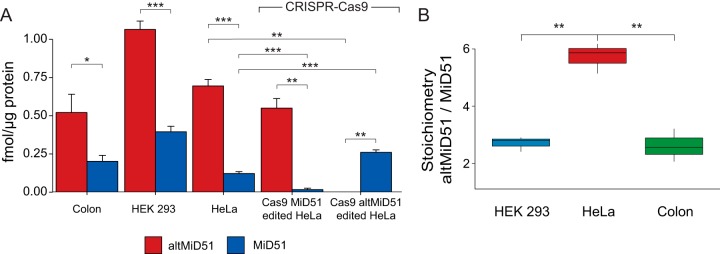
**Absolute quantification and stoichiometries.** *A*, Absolute quantification of altMiD51 and MiD51 in Colon tissue (technical triplicate), HEK 293, HeLa and CRISPR-Cas9 knock outs (biological triplicates). Error bars = standard deviations. *B*, Stoichiometry determination based on absolute quantities of altMiD51 and MiD51. Boxplots represent three biological (HEK 293 and HeLa) or technical (Colon) replicates. Welch's *t* test *p* < 0.05 (*), < 0.01 (**), < 0.001 (***).

AltMiD51 was completely undetectable both by Western blotting (Fig. 3*D*) and absolute quantification (Fig. 4*A*, Welch's *t* test *p* value = 0.0013), confirming successful editing of the altMiD51 ORF (Fig. 3*C*). Remarkably, levels of MiD51 were significantly increased in altMiD51-edited cells (Fig. 4*A*, Welch's *t* test *p* value = 0.0006). Although MiD51 was not detected by Western blotting in CRISPR-Cas9 MiD51 edited cells (Fig. 3*D*), PRM analyses showed a 86% reduction in MiD51 levels (Fig. 4*A*, Welch's *t* test *p* value = 0.0004), suggesting that non-edited WT alleles remained. However, sequencing alleles of MiD51-edited HeLa (Fig. 3*E*) revealed that no WT sequence was detected, suggesting that signal from PRM experiments is because of the 6 nucleotides, and thus 2 amino acids, loss in MiD51 sequence, giving a non-frameshifted sequence coding for a truncated protein containing the AQUA MiD51 peptide (Fig. 3*E*, blue bar). Overall, genome editing of altMiD51 and MiD51 conclusively validated the proteotypic peptides selected for absolute quantification, and the presence of two functional and physically independent coding information in the same gene.

##### Absolute Amounts and Ratio of altMiD51 to MiD51

Absolute quantification performed in HEK 293, HeLa and colon tissue indicate that altMiD51 is the most abundant protein product of *MIEF1* (Fig. 4*A*, supplemental Data S2). We compared absolute quantities of altMiD51 and MiD51 in HEK 293, HeLa and human colon tissue samples to determine their stoichiometric relationship. The stoichiometry indicated that the most abundant translation product from *MIEF1* is altMiD51 rather than the canonical MiD51 protein. The ratio of altMiD51 to MiD51 is 2.71 in HEK 293 cells, 5.73 in HeLa cells, and 2.62 in Human colon tissue (Fig. 4*B* and supplemental Fig. S13). This observation is consistent with our analysis of ribosome occupancy with data extracted from GWIPS (supplemental Fig. S14; supplemental Data S3).

## DISCUSSION

Ribosome profiling and proteogenomics strongly support the translation of alternative protein products from altORFs in addition to the translation of canonical CDSs. Yet, the absolute quantification of a small and a large protein coded by the same gene is unknown. Here, we show that levels of the 70 amino acid altMiD51, a small protein encoded in an exon originally annotated as “non-coding” of *MIEF1/SMCR7L/MiD51* are two to six times higher than the levels of the canonical MiD51 protein in cells and in a human tissue. This work illustrates that small proteins are important contributors of the proteome, and it is not because that altORFs and alternative proteins are not annotated, unlike large proteins, that they do not exist. Obviously, it is very likely that this is not a general feature of altORFs and that the expression levels of small and large proteins coded by the same genes are highly variable and gene-specific. Also, there is no correlation between protein abundance and functionality, and because the ratio altMiD51/MiD51 is > 2 does not mean that the function of altMiD51 is more significant than that of MiD51.

Several physiological processes could explain the higher ratio of altMiD51 to MiD51, including a difference in protein synthesis, a difference in protein degradation or a combination of both. Nonetheless, according to the scanning model for translation initiation, the most likely mechanism is the localization of altMiD51 upstream of MiD51 that would favor altMiD51 translation. This hypothesis is supported by ribosome profiling data aligned to the *MIEF1* locus which indicate that the density of elongating ribosomes is higher on the altORF compared with the CDS (21, 23, 47), suggesting that ribosomes efficiently translate altMiD51. In addition, MIEF1 is moderately resistant to eIF2 repression in response to severe stress induced by sodium arsenite (21). Genes resistant to eIF2 repression are characterized by the presence of an efficiently translated upstream ORF and partial repression of translation of the main CDS in normal conditions, and derepression in response to environmental stresses (56). Our proteomics data agree with the dampening of MiD51 translation under physiological conditions.

CRISPR-mediated altMiD51 and MiD51 KO experiments resulted in two important observations. First, we observed that MiD51 expression was significantly increased in altMiD51 KO cells (Fig. 4*A*). In these cells, the single bp (A/T) insertion in Cas9-edited altMiD51 coding sequence resulted in the truncation of altMiD51 ORF from 210 bps to 78 bps and a parallel increase of intercistronic distance altMiD51-MiD51 from 98 to 231. The combination of a shorter upstream ORF and a longer intercistronic distance were previously shown to increase re-initiation of the downstream ORF (12, 57–60). Thus, in addition to its role as a coding sequence for a novel mitochondrial fission factor (24) and an assembly factor implicated in mitoribosomal biogenesis (25), altMiD51 ORF may function as an upstream ORF regulating the translation of MiD51. Second, MiD51 KO cells still express altMiD51 at normal levels, which demonstrates that knocking out the canonical CDS does not completely inactivate *MIEF1*. This result illustrates for the first time that inactivating an annotated CDS may not necessarily obliterate a gene.

A combination of several circumstances have allowed small proteins to go unnoticed until recently. First, according to current human annotations, protein-coding genes have a single CDS, generally the longest ORF (1). Thus, all efforts to find the physiological function or role in the pathology of a specific gene are invariably focused on the protein encoded by this CDS, or one of its variants generated by alternative splicing. Second, in the absence of annotation of non-canonical ORFs, the protein sequence of the corresponding proteins cannot be routinely detected by MS-based proteomics approaches which rely on current protein databases containing the sequences of canonical proteins only. Third, the widely used Western blotting technique relies on specific antibodies, but antibodies have been raised and commercialized for canonical proteins only. Raising novel specific antibodies may take time and several attempts, thus delaying the investigations on small proteins. Fourth, the detection of small proteins by MS-based proteomics is more challenging than for large proteins. Typically, the proteome has to be fractionated to enrich low molecular weight proteins, and the identification often relies on a single tryptic peptide (5, 9). In addition, there may be no sites for trypsin digestion and peptides exceeding 25 aa are rarely identified in bottom-up proteomics. Fifth, because they are short, small proteins are less likely to have known protein domains discovered in large proteins, or to display a specific structure. Thus, there might exist a biased perception that small proteins have minor functions compared with large proteins in biological mechanisms. Yet, many small proteins have essential functions in prokaryotes and eukaryotes (14, 61).

AltMiD51 was integrated into the automatically annotated UniProtKB/TrEMBL database (identifier L0R8F8) in March 2013, following its detection under the name altSMCR7L (3). It was integrated into the manually annotated UniProtKB/Swiss-Prot database in March 2017. Like altMiD51, which is now a manually annotated bicistronic gene, it will be important to update genome annotations according to recent proteogenomics studies (14, 24). Indeed, the function of a dual-coding gene should not be inferred according to the molecular activity of the larger protein product only. In addition, the impact of mutations on gene function should not be analyzed in the conceptual frame of a single CDS, because mutations outside currently annotated CDSs may affect noncanonical ORFs and ultimately, gene function (62). Finally, our results clearly demonstrate that knocking out the canonical CDS in a gene and leaving altORFs unnaltered does not completely abrogate the translation output of that gene.

## DATA AVAILABILITY

Mass spectrometry data are available at the PRIDE repository with the data set identifier PXD008147.

## Supplementary Material

supplemental Data S1

## Footnotes

^1^ The abbreviations used are:

ORF: open reading frames.
