# Supplementary material for: The Protein Coded by a Short Open Reading Frame, Not by the Annotated Coding Sequence, Is the Main Gene Product of the Dual-Coding Gene MIEF1
Source: Mol Cell Proteomics. 2018 Sep 4;17(12):2402–11. doi: 10.1074/mcp.RA118.000593 (PMC6283296; doi:10.1074/mcp.RA118.000593)
Supplement: supplemental Data S1 [file RA118.000593_index.html]

Supplement to The protein coded by a short open reading frame, not by the annotated coding sequence is the main gene product of the dual-coding gene MIEF1 | Molecular & Cellular Proteomics

## Supplemental Data

- Supplementary figures - Supplementary figures
- Supplementary data 1 - Proteotypic peptides for MiD51 and altMiD51
- Supplementary data 2 - Absolute quantification of peptides
- Supplementary data 3 - Ribosome profiling for altMiD51 and MiD51
