# Supplementary material for: The Protein Coded by a Short Open Reading Frame, Not by the Annotated Coding Sequence, Is the Main Gene Product of the Dual-Coding Gene MIEF1
Source: Mol Cell Proteomics. 2018 Sep 4;17(12):2402–11. doi: 10.1074/mcp.RA118.000593 (PMC6283296; doi:10.1074/mcp.RA118.000593)
Supplement: supplemental Data S1 [file 135144_1_supp_167624_pc4m86.pdf]

Supplementary figures

First evidence that a protein coded by a short ORF, not by the annotated CDS is the main gene product

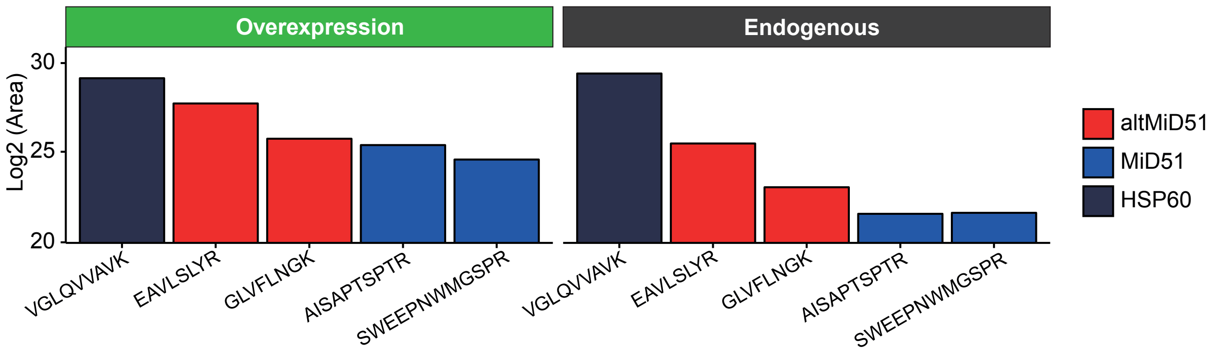

Supplementary Figure 1 : Proteotypic peptides validation by low sensitivity PRM in mitochondrial extracts in transfected and endogenous HeLa cells. Endogenous HSP60 is used here as a control in both experiments.

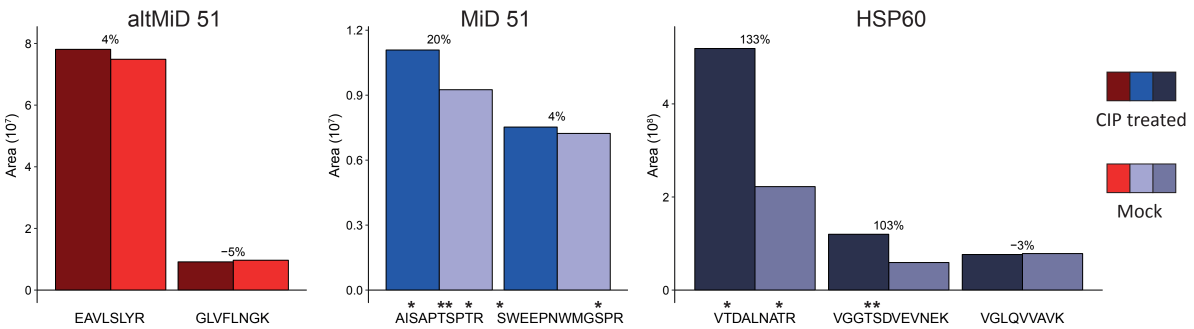

Supplementary Figure 2 : Evaluation of CIP treatments for dephosphorylation of peptides. For each peptide, signal variation are indicated as percentage on top of histograms. * : known phosphorylated residue (phosphosite.org)

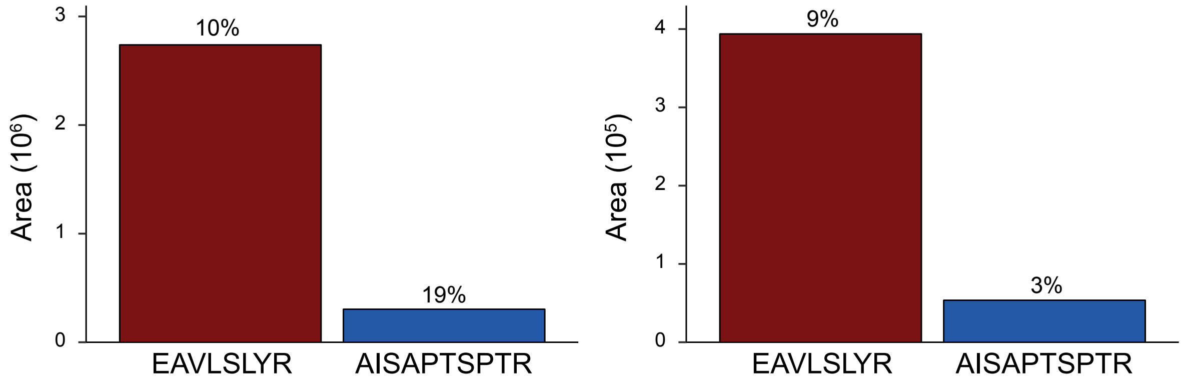

Supplementary Figure 3 : CV analysis of endogenous altMiD51 (red) and MiD51 (blue) in mitochondrial extracts (left) and whole cell lysates (right). CV are indicated on top of histograms.

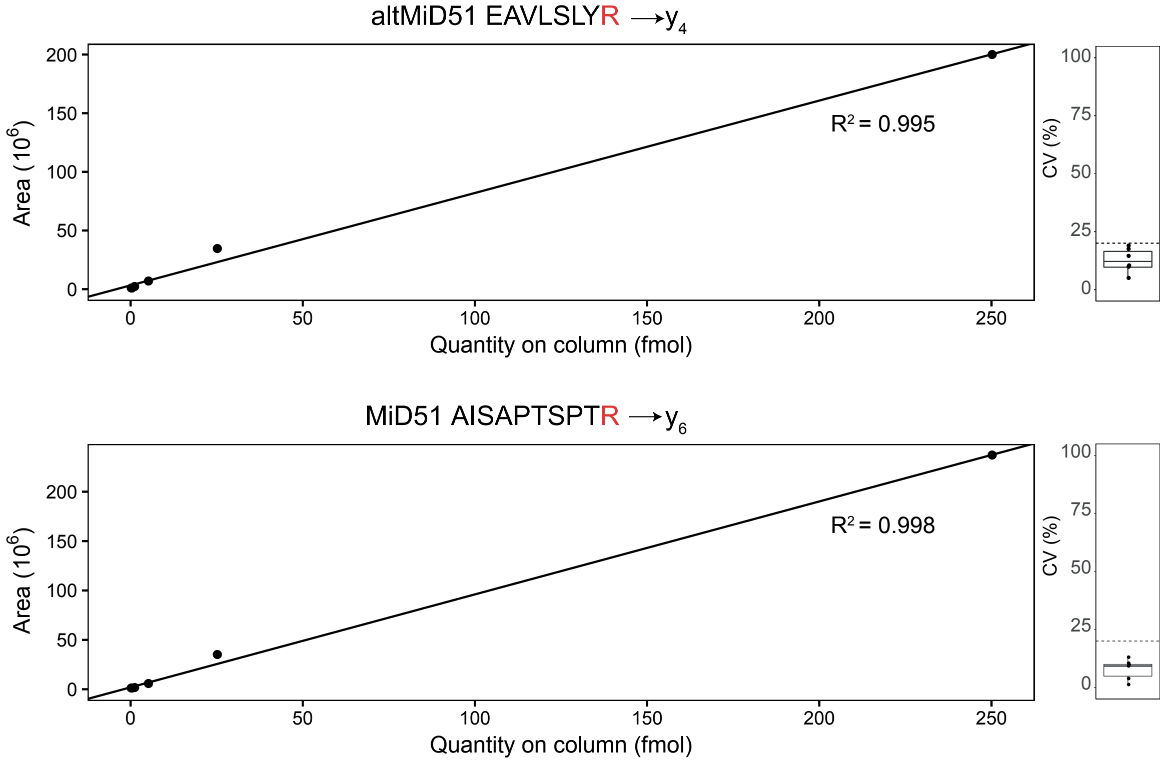

Supplementary Figure 4 : Linear regression and CV analysis of stable isotope labeled peptides.

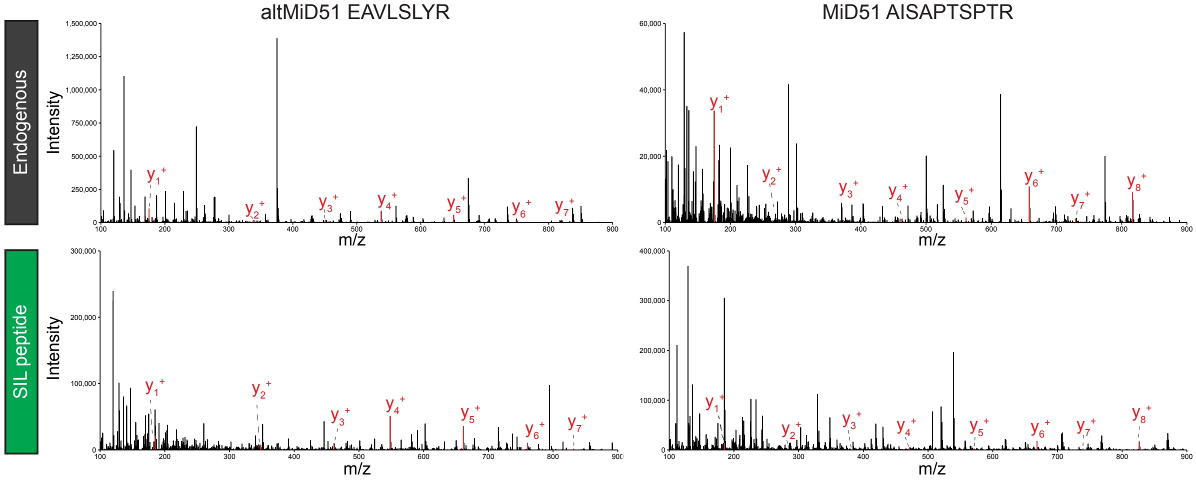

Supplementary Figure 5 : PRM MS/MS spectra of EAVLSLYR and AISAPTSPTR peptides and their stable isotope labeled peptides in a HeLa sample.

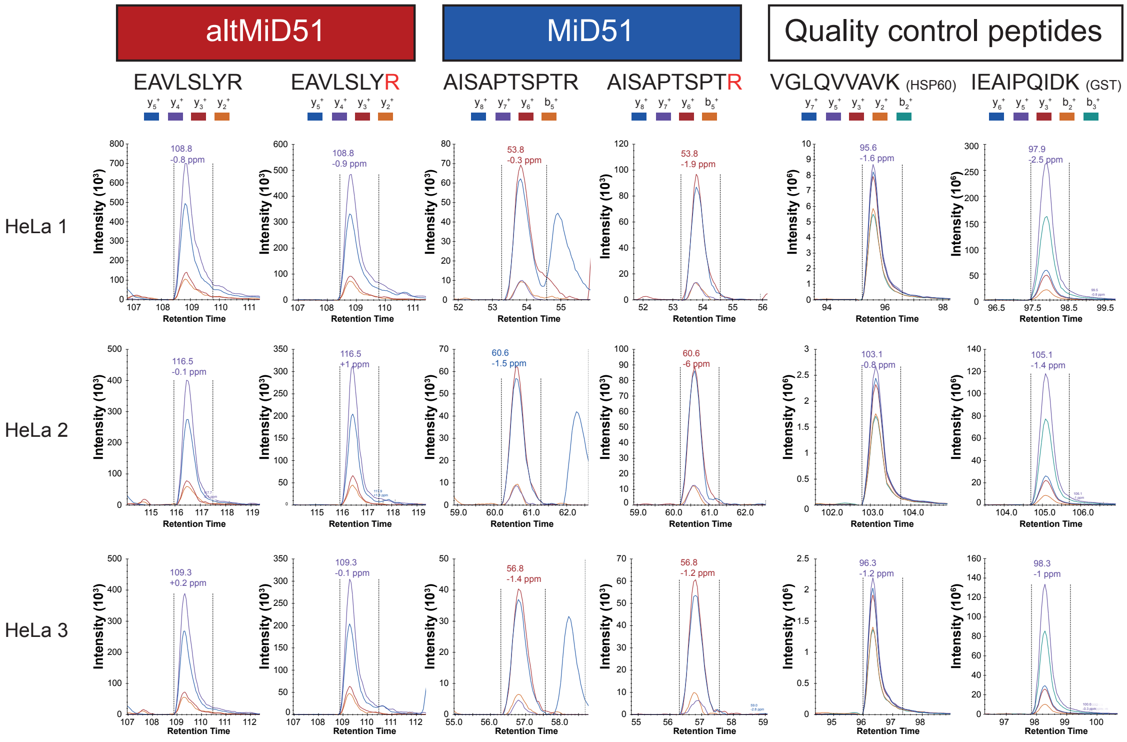

Supplementary Figure 6 : Fragment ion traces in HeLa samples.

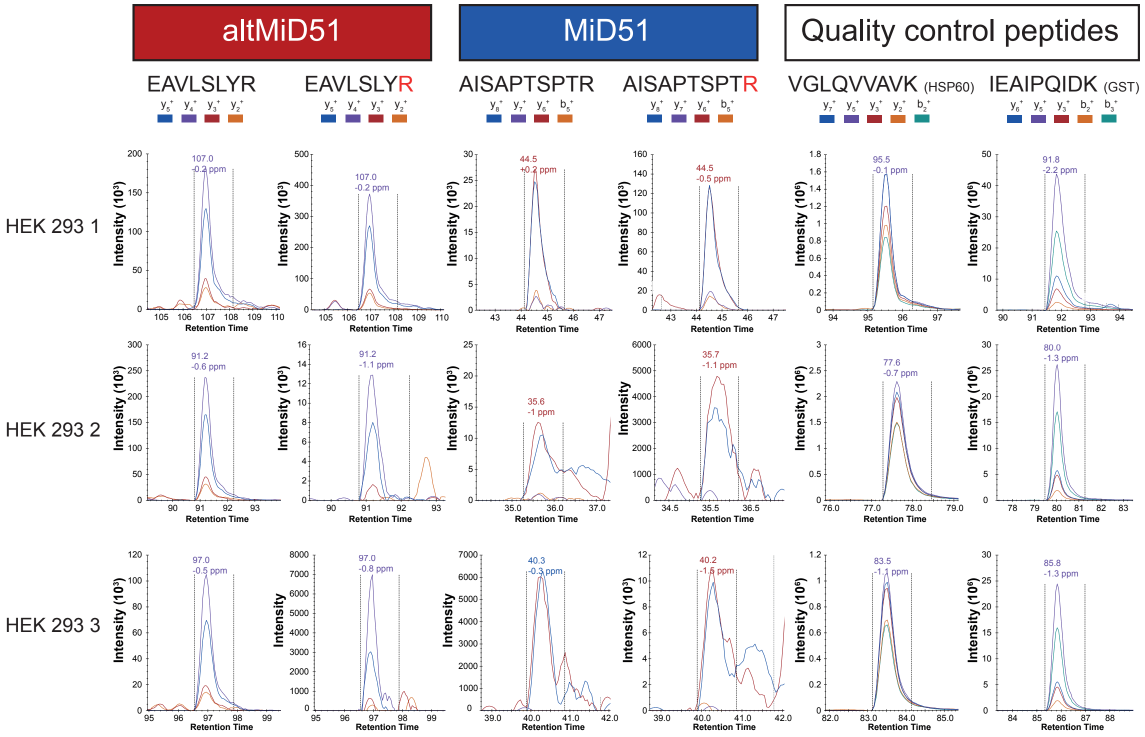

Supplementary Figure 7 : Fragment ion traces in HEK 293 samples.

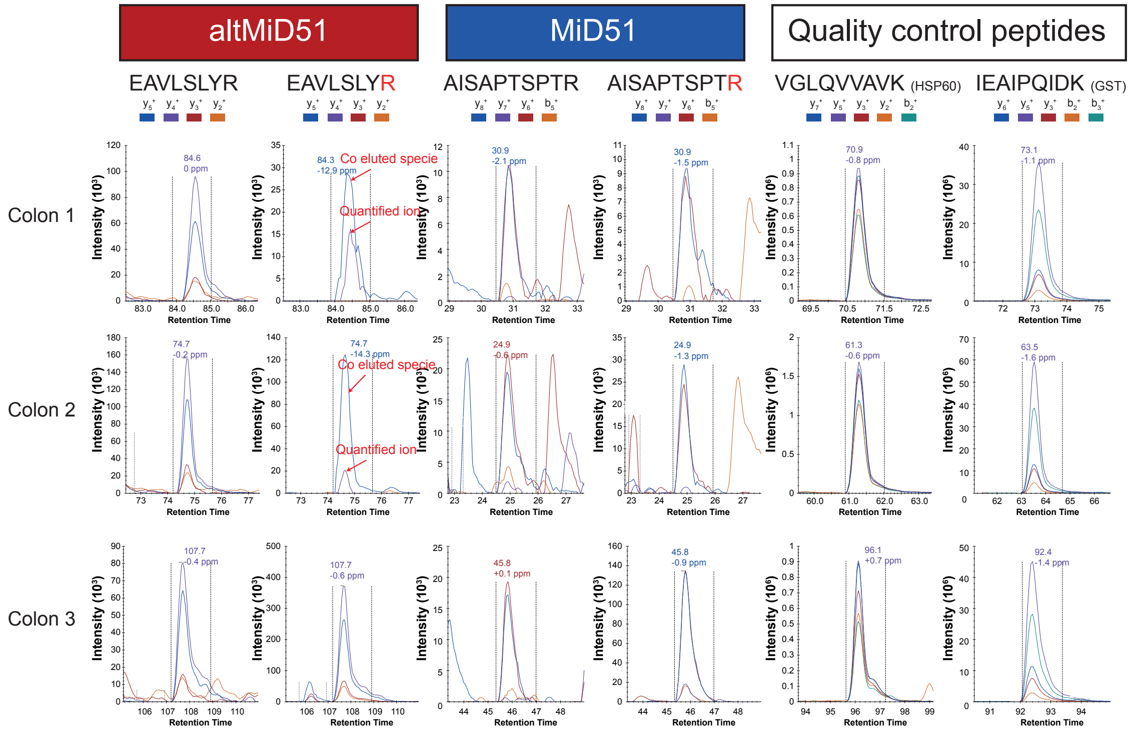

Supplementary Figure 8 : Fragment ion traces in colon tissue samples.

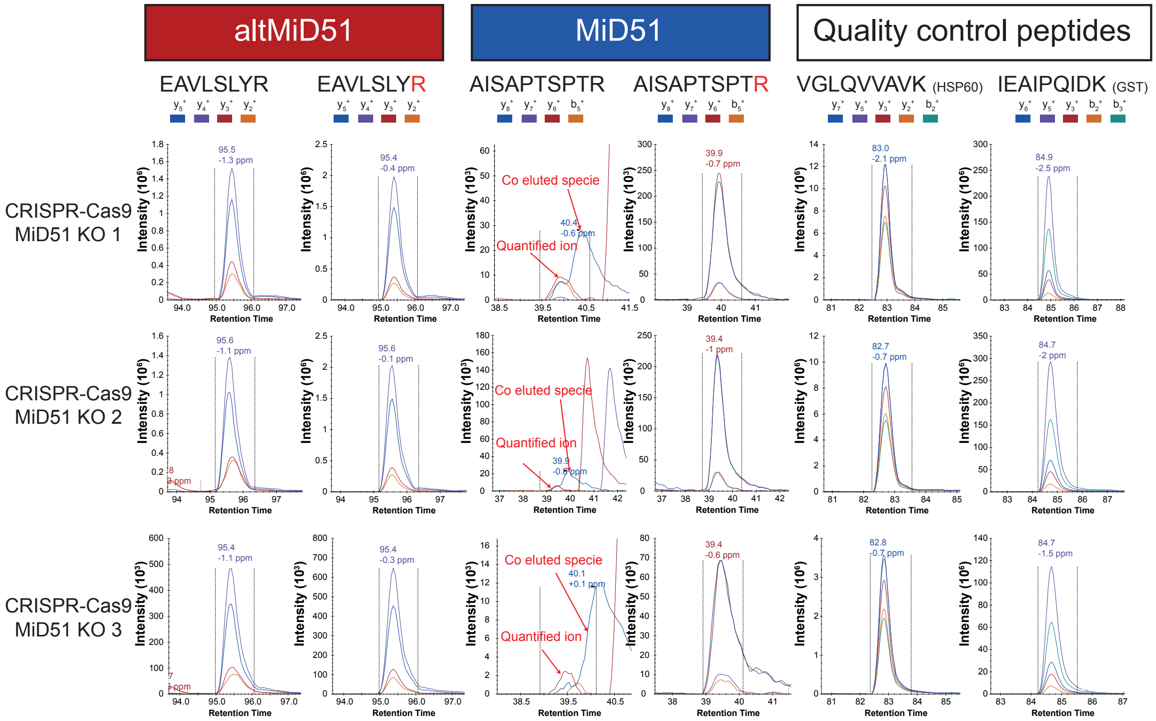

Supplementary Figure 9 : Fragment ion traces in CRISPR-Cas9 MiD51 edited HeLa samples.

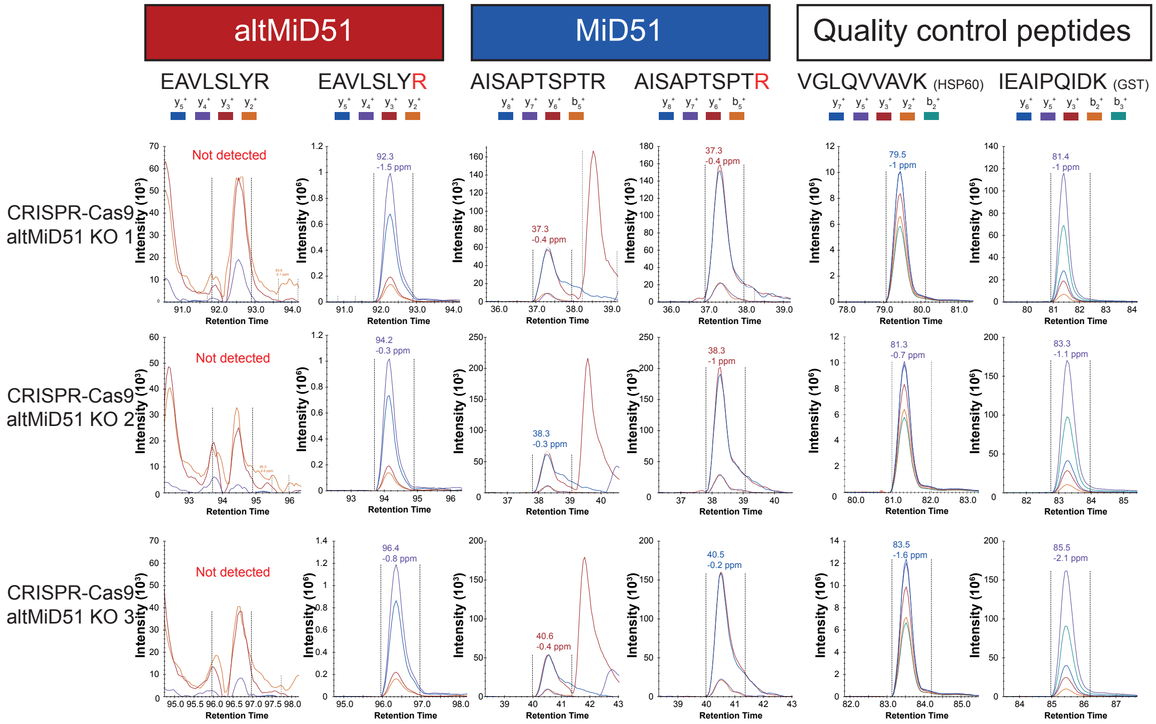

Supplementary Figure 10 : Fragment ion traces in CRISPR-Cas9 altMiD51 edited HeLa samples.

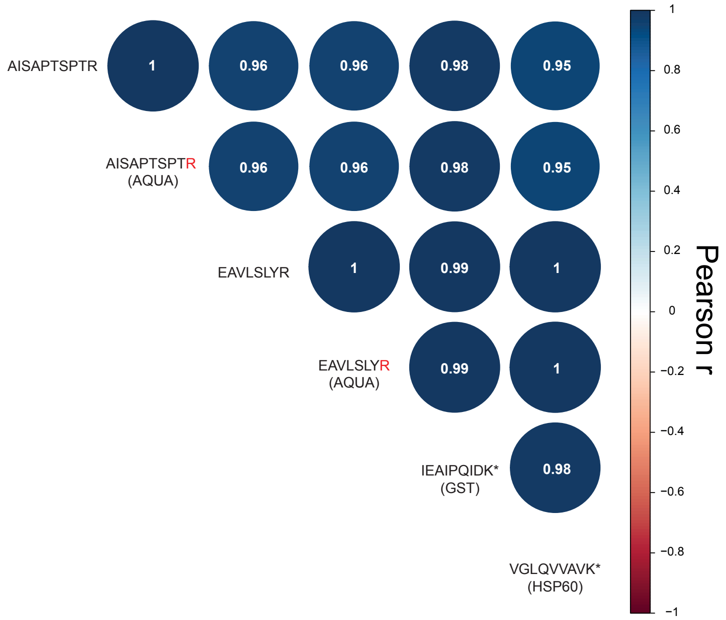

Supplementary Figure 11 : Peptide retention time correlation across samples. Circle color and size indicate Pearson r value which is reported inside each circle. CRISPR-Cas9 altMiD51-edited samples were excluded as altMiD51 endogenous peptide EAVLSLYR was not detected. * Peptides from GST and HSP60 were used as sample-processing controls.

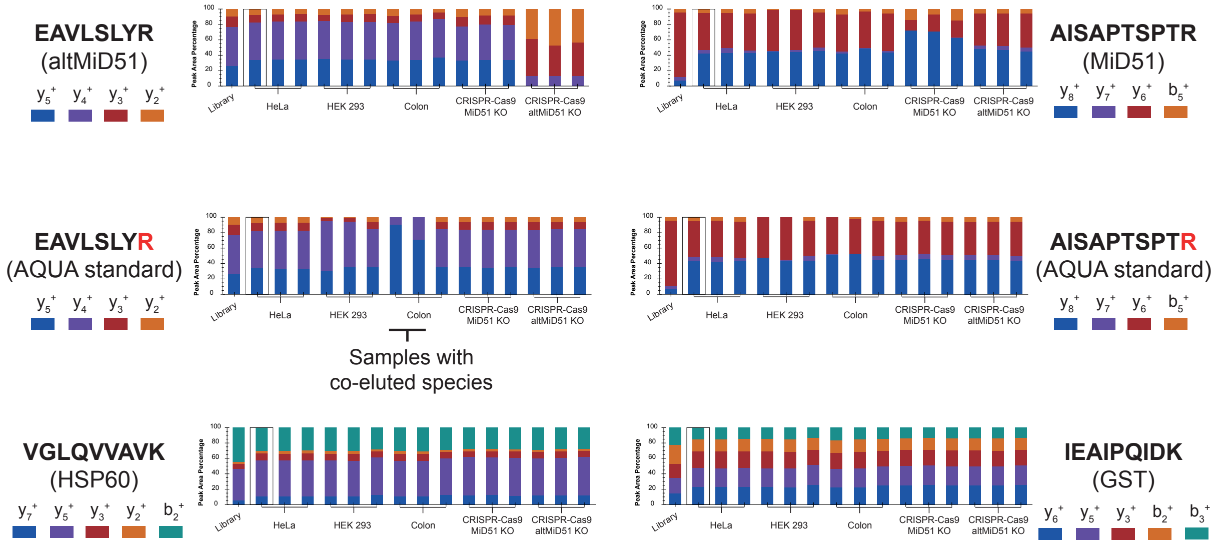

Supplementary Figure 12 : Peptides fragmentation relative intensities across samples in absolute quantification PRM experiments which were used to ensure correct identification of peptides.

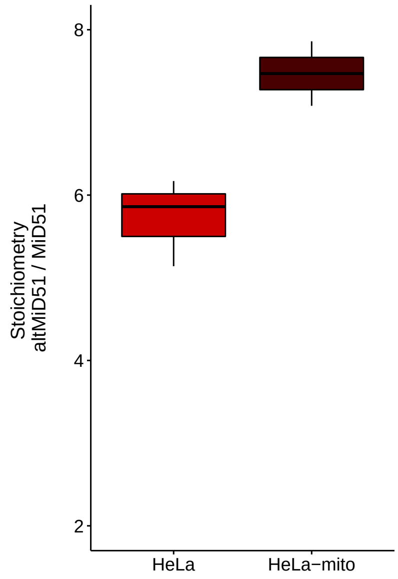

Supplementary Figure 13 : Stoichiometry comparison between HeLa whole cells extracts (biological triplicate) and HeLa mitochondria (technical duplicate).

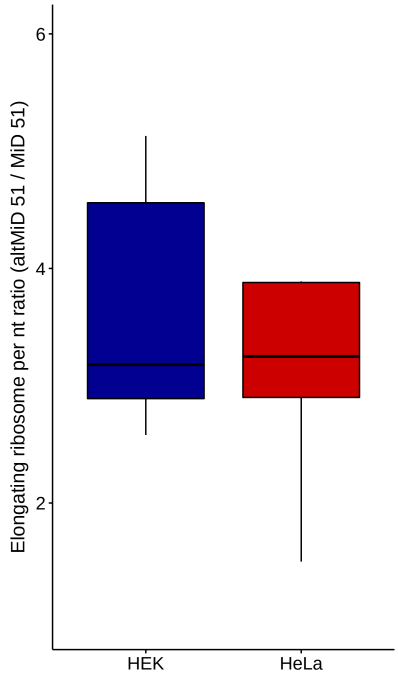

Supplementary Figure 14 : Elongating ribosomes ratio in HEK 293 and HeLa cell lines where the total number of elongating ribosomes per mappable nucleotide (nt) for the altMiD51 ORF was compared to elongating ribosomes per mappable nt for the MiD51 ORF.
